# Effect of coadministration of rifampicin on the pharmacokinetics of linezolid: clinical and animal studies

**DOI:** 10.1186/s40780-018-0123-1

**Published:** 2018-11-12

**Authors:** Satsuki Hashimoto, Kyoko Honda, Kohei Fujita, Yuka Miyachi, Kazuya Isoda, Ko Misaka, Yukio Suga, Satoshi Kato, Hiroyuki Tsuchiya, Yukio Kato, Masaki Okajima, Takumi Taniguchi, Tsutomu Shimada, Yoshimichi Sai

**Affiliations:** 1 Department of Hospital Pharmacy, University Hospital, Kanazawa University, 13-1, Takara-machi, Kanazawa, Ishikawa 920-8641 Japan; 2 Department of Medicinal Informatics, Graduate School of Medical Sciences, Kanazawa University, 13-1, Takara-machi, Kanazawa, Ishikawa 920-8641 Japan; 3 Faculty of Pharmacy, Institute of Medical, Pharmaceutical and Health Science, Kanazawa University, Kakuma-machi, Kanazawa, Ishikawa 920-1192 Japan; 4 Department of Orthopaedic Surgery, Graduate School of Medical Sciences, Kanazawa University, 13-1 Takara-machi, Kanazawa, Ishikawa 920-8641 Japan; 5 Intensive Care Unit, University Hospital, Kanazawa University, 13-1 Takara-machi, Kanazawa, Ishikawa 920-8641 Japan

**Keywords:** Linezolid, Rifampicin, Drug-drug interaction, Therapeutic drug monitoring, Pharmacokinetics, Adverse event

## Abstract

**Background:**

Combination therapy of linezolid (LZD) and rifampicin (RFP) may be more effective than monotherapy for treating gram-positive bacterial infections, but several studies have suggested that RFP decreases LZD exposures, thereby increasing the risk of therapeutic failure and emergence of LZD-resistant strains. However, the mechanism of the drug-drug interaction between LZD and RFP is unknown.

**Methods:**

We conducted a prospective, open-label, uncontrolled clinical study in Japanese patients receiving LZD and RFP to evaluate the effect of coadministered RFP on the concentration of LZD. In animal study in rats, the influence of coadministered RFP on the pharmacokinetics of LZD administered intravenously or orally was examined. Intestinal permeability was investigated with an Ussing chamber to assess whether coadministered RFP alters the absorption process of LZD in the intestine.

**Results:**

Our clinical study indicated that multiple doses of RFP reduced the dose-normalized trough concentration of LZD at the first assessment day by an average of 65%. In an animal study, we found that multiple doses of RFP significantly decreased the area under the concentration-time curve, the maximum concentration and the bioavailability of orally administered LZD by 48%, 54% and 48%, respectively. In contrast, the pharmacokinetics of intravenously administered LZD was unaffected by the RFP pretreatment. However, investigation of the intestinal permeability of LZD revealed no difference in absorptive or secretory transport of LZD in the upper, middle and lower intestinal tissues between RFP-pretreated and control rats, even though RFP induced gene expression of multidrug resistance protein 1a and multidrug resistance-associated protein 2.

**Conclusions:**

Therapeutic drug monitoring may be important for avoiding subtherapeutic levels of LZD in the combination therapy. The drug-drug interaction between LZD and RFP may occur only after oral administration of LZD, but is not due to any change of intestinal permeability of LZD.

**Trial registration:**

UMIN, UMIN000004322. Registered 4 October 2010.

## Background

Linezolid (LZD) is an oxazolidinone antimicrobial agent with broad-spectrum activity against gram-positive bacteria, including methicillin-resistant *Staphylococcus aureus* (MRSA) and vancomycin-resistant *Enterococcus faecium* [1]. It is rapidly absorbed after oral administration, with 100% bioavailability (F), and is metabolized by nonenzymatic oxidation into two inactive metabolites, without the involvement of any major cytochrome P450 (CYP) [2–4]. About 30% of LZD is eliminated in unchanged form in the urine, and the major metabolites are also mainly excreted via the kidney [4]. On the other hand, the mechanisms involved in the permeability of LZD in small intestines have not been fully clarified.

Early studies suggested that therapeutic drug monitoring (TDM) and dose adjustment based on body weight might be unnecessary during LZD therapy. However, some recent studies have indicated that major adverse events associated with LZD, particularly thrombocytopenia and anemia, could appear dose-dependently [5–9]. Moreover, there are several reports of drug-drug interactions (DDI) with LZD in humans: coadministration of omeprazole, amiodarone, amlodipine, sertraline or clarithromycin with LZD increased the exposure to LZD  [9–11]. It was speculated that P-glycoprotein (P-gp) could be involved in these DDIs, because omeprazole, amiodarone, amlodipine, sertraline or clarithromycin are known to be P-gp inhibitors. On the other hand, it has been reported that rifampicin (RFP) decreases LZD exposure in terms of trough concentration (C_min_), maximum concentration (C_max_) and area under the concentration-time curve (AUC), and RFP also reduced the incidence of LZD-induced thrombocytopenia and/or anemia [12–15]. The mechanism of the DDI between LZD and RFP remains unknown. The various DDIs with LZD may result in marked inter-individual variability in LZD exposure and concentration-dependent adverse events. Thus, it seems necessary to monitor LZD concentration during administration, and also to identify the mechanisms involved in these DDIs.

The aim of the present study was: (i) to evaluate the effect of coadministered RFP on the concentration of LZD in a prospective, open-label, uncontrolled clinical study, (ii) to determine the influence of coadministered RFP on the pharmacokinetics of LZD administered intravenously or orally in rats, and (iii) to assess whether coadministered RFP alters the absorption process of LZD in the intestine.

## Methods

### Materials

LZD injection solution (Zyvox Injection, 600 mg) used for intravenous administration and tablets (Zyvox Tablets, 600 mg) used for oral administration in rats were purchased from Pfizer Japan, Inc. (Tokyo, Japan). RFP, Lucifer yellow (LY) and rhodamine 123 (Rho123) were purchased from Sigma-Aldrich (Tokyo, Japan). Pentobarbital and diethyl ether were purchased from Nakalai Tesque, Inc. (Kyoto, Japan). All other chemicals used were of analytical or high performance liquid chromatography (HPLC) grade.

### Subjects and study design

This prospective, open-label, uncontrolled study was conducted from October 2010 through October 2013 at Kanazawa University Hospital. The study was approved (UMIN000004322) by the ethics committee of Kanazawa University Hospital, and written informed consent was obtained from all participants, who were adults (≥ 20 years) being treated with oral LZD 600 mg every 12 h. Patients who were treated with RFP after the initiation of LZD therapy were excluded from the study. The main reasons for LZD treatment were orthopedic device-related infections, and bone and joint infections. Microbiological isolates were identified in 90% of cases, and were mainly MRSA and *Staphylococcus epidermidis*. None of the isolates was resistant to LZD according to the Clinical and Laboratory Standards Institute criteria, and all of the isolates had an MIC ≤2 μg/mL.

Patients were divided into two subgroups. One (the LZD/RFP group) received oral coadministration of RFP 450 mg every 24 h for 3–15 days, while the other (the LZD group) did not receive RFP coadministration. At the first assessment day (on days 2–5 after the initial administration), blood was sampled just before the subsequent administration of LZD to measure the trough concentration. The blood was centrifuged, and the serum was stored at − 30 °C until analysis.

Thrombocytopenia was defined as a decrease in the platelet count to < 130,000 /μL, and anemia was defined as a decrease in the hemoglobin (Hb) concentration to < 8 g/dL. We determined fourteen variables: sex, age, body weight, estimated glomerular filtration rate (eGFR), C-reactive protein (CRP), platelet count, Hb concentration, duration of LZD therapy, total dosage, daily dose, trough concentration of LZD at the first assessment day during LZD therapy, number of instances of TDM, concomitant drugs received during LZD therapy, and success rate. Dose adjustment of LZD was performed to avoid LZD-related adverse events. eGFR was estimated based on the Clinical Practice Guidebook for Diagnosis and Treatment of Chronic Kidney Disease.

After an average follow-up of 2.4 years, patients were considered cured if there was no clinical, biological or radiological evidence of infection. In other cases, treatment was considered to have failed.

### Animal experiments

Male Sprague-Dawley rats (10 weeks old) were purchased from Japan SLC, Inc. (Hamamatsu, Japan). Rats were housed under a 12-h light, 12-h dark cycle and were fed normal diet and water *ad libitum*. Rats were acclimated for 1 week before drug administration. In the RFP pretreatment study, RFP dissolved in 1% (w/v) CMC-Na was administered orally once a day at 10 mg/kg for 4 days [16]. Control rats received 1% (w/v) CMC-Na orally. Following these treatments, rats were given a single dose of LZD orally (62.5 mg/kg) via a gastric tube or intravenously (45.7 mg/kg) from the jugular vein at 12 h after the last RFP administration, under anesthesia with diethyl ether. Blood samples (250 μL each) were collected prior to dosing of LZD and at 0.25, 0.5, 0.75, 1, 2, 3, 6 and 12 h after dosing from the opposite jugular vein, and were centrifuged to obtain plasma. All animal procedures were carried out in accordance with the Guidelines for the Care and Use of Laboratory Animals at Kanazawa University.

### Measurement of LZD by LC/MS

LZD was quantitated by means of validated liquid chromatography-mass spectrometry (LC/MS) according to the procedure of Slatter et al., with minor modifications [4]. In brief, plasma samples (100 μL) were mixed with acetonitrile (100 μL) for 10 min in a shaker and then centrifuged at 10,000×g for 5 min at 4 °C. An aliquot of the supernatant (20 μL) was analyzed to determine the LZD concentration. Separation was done on a Symmetry C8 column (250 × 4.6 mm, 5 μm; Waters, Co., Tokyo, Japan) using an isocratic mobile phase of 100 mM ammonium acetate (pH 4.8) / acetonitrile (75:25, v/v) at a flow rate of 1.0 mL/min. The calibration plot was linear over the range of 0.5 to 50 μg/mL with a correlation coefficient ≥ 0.99. The intra- and inter-assay coefficients of variation were all < 10%. The lower limit of detection was 0.5 μg/mL. Pharmacokinetic parameters were estimated by model-independent moment analysis, including AUC, C_max_, elimination rate constant (k_e_), half-life (t_1/2_), total clearance (CL_tot_), volume of distribution (V_d_) and F.

### RNA extraction and real-time PCR

After euthanasia, the entire length of the rat small intestine was quickly removed. Intestinal segments were isolated and each site was defined as described below [17]. A 5 cm portion of the top of the small intestine was regarded as the duodenum (the upper intestine). The ileum (the lower intestine) was obtained from the final 5 cm portion of the intestine. The jejunum (the middle intestine) was obtained from the remaining portion. Each intestinal segment was snap-frozen in liquid nitrogen and stored at − 80 °C until analysis. Total RNA was extracted using a GenElute™ Mammalian Total RNA Miniprep Kit (Sigma-Aldrich, Tokyo, Japan), according to the manufacturer’s protocol. The concentration of total RNA was measured with a NanoDrop® ND-1000 spectrophotometer (NanoDrop Products, Wilmington, DE, USA). cDNA was synthesized with 2 μg of total RNA using a High Capacity cDNA Reverse Transcription Kit® (Applied Biosystems, Foster city, CA, USA), according to the manufacturer’s instructions. To prepare a standard curve, cDNA was mixed with Platinum® PCR SuperMix (Invitrogen Life Technologies Japan Ltd., Tokyo, Japan), and amplified by using a Gene Amp® PCR System 9700 (Applied Biosystems, Foster city, CA, USA). The PCR conditions were 35 cycles at 94 °C for 15 s, 60 °C for 15 s and 72 °C for 30 s. PCR products were separated on a 2% agarose gel. For gene expression studies, the cDNA was mixed with THUNDERBIRD® SYBR® qPCR Mix (Toyobo Co., Ltd., Osaka, Japan) and gene-specific primers (Invitrogen Life Technologies Japan Ltd., Tokyo, Japan). The primers used were as follows: multidrug resistance protein 1a (Mdr1a/Abcb1a), 5’-TGAACTGTGACCATGCGAGATGTTAAATA-3′ and 5’-GTCTCTGAAGACTCTAAAATGGACTAAATG-3′ for a 153-bp fragment; multidrug resistance-associated protein 2 (Mrp2/Abcc2), 5’-TTCACGGGCACATCACCA-3′ and 5’-ATTCGGACCCAAACAGGATG-3′ for a 102-bp fragment; breast cancer resistance protein (Bcrp/Abcg2), 5’-GTTTGGACTAAGCACAGCA-3′ and 5’-TGAGTTTCCCAGAAGCCAGT-3′ for a 150-bp fragment; and β-actin, 5’-TGAGCGCAAGTACTCTGTGTGGAT-3′ and 5’-TAGAAGCATTTGCGGTGCACGATG-3′ for a 129-bp fragment. The PCR conditions for the Mx3000P® Real-Time QPCR System 9700 (Agilent Technologies, Inc., Santa Clara, CA, USA) were 40 cycles at 95 °C for 30 s and 60 °C for 60 s.

### Measurement of intestinal permeability in an Ussing chamber

Intestinal permeability study was examined by means of the Ussing chamber technique, as described in the literature [18]. Rho123, inulin, LZD and LY were dissolved at concentrations of 5 μM, 5 mg/mL, 20 μg/mL and 5 μM, respectively, in buffer solution (pH 7.0) composed of 1.4 mM CaCl_2_, 5.1 mM KCl, 1.3 mM KH_2_PO_4_, 1.3 mM MgSO_4_∙7H_2_O, 128 mM NaCl, 10 mM NaH_2_PO_4_∙2H_2_O, 5 mM D-glucose, and 21 mM NaHCO_3_. Inulin and LY were used as paracellular permeability markers. Rats with/without RFP pretreatment for four days as above were fasted for 24 h, then anesthetized with sodium pentobarbital (30 mg/kg, i.p.). The upper, middle, lower intestine as defined above, were collected. Segments were cut open, the muscle layer was stripped, and the intestinal sheets were mounted in Ussing chambers (Sakuma, Tokyo, Japan) with an exposed area of 0.5 cm^2^. Each side of the tissue was bathed with buffer solution (2 mL) under CO_2_/O_2_ (5%/95%). The entire assembly was maintained at 37 °C. During the transport studies, 0.2 mL aliquots were taken from the receiver side at 0, 30, 45, 60, 90, 120 and 180 min, and immediately replaced with an equal volume of buffer solution. The amount of Rho123 in the receiver side was assayed by HPLC according to the method of Cho et al., with minor modifications [19]. Concentrations of inulin were colorimetrically determined as described in the literature [20]. Concentrations of LZD were measured as described above and LY was measured by HPLC according to the method of Lin et al. [17]. Standard calibration curves were constructed for each compound within appropriate concentration ranges. In all cases, the curves displayed excellent linearity with r^2^ > 0.99.

Apparent permeability coefficients (P_app_) of Rho123, inulin, LZD, and LY in cm/s, were calculated as follows:

$$ {\mathrm{P}}_{\mathrm{app}}={\mathrm{P}}_{\mathrm{amount}}/\left({\mathrm{C}}_0\bullet \mathrm{A}\bullet \mathrm{t}\right) $$

where P_amount_ (μmol) is the total amount of drug that permeated to the receiver side throughout the incubation time, C_0_ (μmol/mL) is the drug concentration before transport on the donor side, A (cm^2^) is the area of the diffusion chamber for transport, and t (s) is the experimental duration. The efflux ratio (ER) was obtained as (P_app_, _s-m_/P_app_, _m-s_), where P_app_, _m-s_ is P_app_ of absorption (mucosal to serosal, m-s) and P_app_, _s-m_ is P_app_ of secretion (serosal to mucosal, s-m).

### Statistical analysis

Values are expressed as mean ± SD. Statistical comparisons were performed by means of an unpaired Student’s *t*-test. A value of *p* < 0.05 was considered to indicate statistical significance.

## Results

### Effects of RFP coadministration on LZD concentration, adverse events and outcomes in patients

The present study included 7 patients in the LZD group and 3 patients in the LZD/RFP group. The characteristics, LZD therapy, adverse events and outcomes of the patients are shown in Table 1. No drug known to show DDI with LZD was administered during LZD therapy [9–12].

**Table 1:** Baseline characteristics and clinical outcomes of patients

	LZD group		LZD/RFP group	
Gender (male/female)	4 / 3		2 / 1	
Age (year)	60 ± 19	[21–82]	51 ± 11	[41–62]
Body weight (kg)	66.0 ± 17.2	[46.6–95.0]	57.8 ± 14.5	[46.0–74.0]
eGFR (mL/min/1.73 m^2^)	65.2 ± 26.3	[32.7–105]	99.9 ± 34.7	[65.6–135]
Baseline CRP concentration (mg/dL)	3.0 ± 3.1	[0.7–8.9]	1.8 ± 0.6	[1.2–2.4]
LZD dose and concentrations				
Total dose (g)	14.3 ± 5.2	[7.2–21.0]	28.4 ± 9.1	[18.0–34.8]
Daily dose (g/day)	1.04 ± 0.21	[7.29–1.20]	1.20 ± 0.0	
Daily dose (mg/kg/day)	16.7 ± 5.7	[10.5–25.8]	21.6 ± 5.0	[16.2–26.1]
Duration of LZD therapy (day)	14 ± 6	[6–21]	24 ± 8	[15–29]
Number of TDM	21		13	
Trough concentration at first assessment day (μg/mL)	13.3 ± 8.4	[4.7–29.4]	6.9 ± 5.0	[2.1–12.1]
C/D ratio at first assessment day (μg/mL/mg/kg/day)	0.83 ± 0.46	[0.32–1.34]	0.29 ± 0.17	[0.13–0.47]
Patients with dosage adjustments to avoid overexposure, n (%)	3 (42.9%)		0 (0%)	
CRP concentration at first assessment day (mg/dL)	4.2 ± 4.6	[0.3–13.8]	2.0 ± 2.0	[0.7–4.3]
Hematological adverse effects				
Baseline platelet count (10^6^ platelets/μL)	266 ± 88	[174–445]	325 ± 128	[189–443]
Nadir platelet count (10^6^ platelets/μL)	150 ± 82	[86–320]	173 ± 113	[69–293]
Thrombocytopenia, n (%)	4 (57.1%)		1 (33.3%)	
Baseline Hb (g/dL)	9.6 ± 1.8	[7.3–12.9]	11.4 ± 1.2	[10.1–12.2]
Nadir Hb (g/dL)	8.9 ± 2.2	[6.8–12.8]	10.4 ± 2.8	[7.1–12.1]
Anemia, n (%)	3 (42.9%)		1 (33.3%)	
Outcomes				
Success, n (%)	5 (71.4%)		3 (100%)	

*C/D ratio* dose-normalized trough concentration, *Hb* hemoglobin, *eGRF* estimated glomerular filtration rate, *CRP* C-reactive protein

Coadministration with RFP reduced the dose-normalized trough concentration (C/D ratio) of LZD at the first assessment day by an average of 64.7%.

### Effects of RFP pretreatment on the pharmacokinetics of LZD after intravenous and oral administration of LZD to rats

**Fig. 1:**
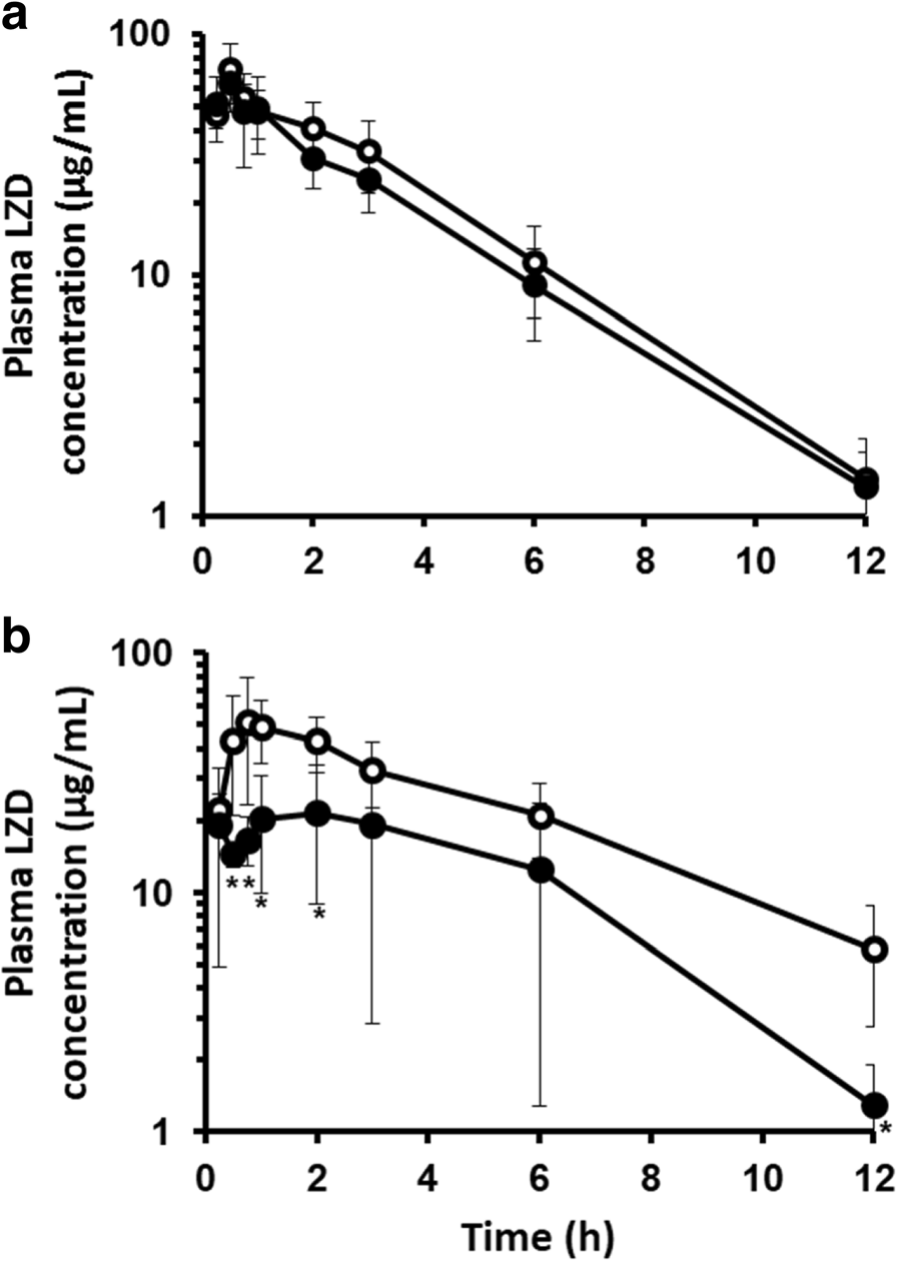
Plasma concentration-time profiles of LZD after intravenous and oral administration with and without RFP. **a** Plasma concentration-time profiles of LZD after intravenous administration of LZD (45.7 mg/kg) to rats with (closed circles) and without (open circles) RFP pretreatment (10 mg/kg) for four days. **b** Plasma concentration-time profiles of LZD after oral administration of LZD (62.5 mg/kg) to rats with (closed circles) and without (open circles) RFP pretreatment (10 mg/kg) for four days. Values are mean ± SD of three to six rats. *p < 0.05

When LZD was administered intravenously to rats pretreated with RFP for four days, the RFP pretreatment had no effect on the plasma concentration-time profile or the pharmacokinetic parameters of LZD (Fig. 1a, Table 2). In contrast, when LZD was administered orally to RFP-pretreated rats, RFP significantly decreased the plasma concentration of LZD (Fig. 1b), and the AUC, C_max_ and F of LZD were significantly reduced by approximately 48.1%, 53.9% and 48.1% (Table 2).

**Table 2:** Pharmacokinetic parameters of LZD after intravenous (i.v.) and oral (p.o.) administration with and without RFP

			Control	with RFP
i.v.	AUC_0–12_	μg/mL ▪ h	232 ± 61	194 ± 54
	C_max_	μg/mL	72.5 ± 17.8	65.9 ± 15.2
	k_e_	h^−1^	0.292 ± 0.041	0.329 ± 0.048
	t_1/2_	h	2.41 ± 0.33	2.14 ± 0.29
	CL_tot_	L/h/kg	0.210 ± 0.064	0.253 ± 0.078
	V_d_	L/kg	0.693 ± 0.156	0.758 ± 0.262
p.o.	AUC_0–12_	μg/mL ▪ h	280 ± 64	145 ± 103*
	C_max_	μg/mL	48.0 ± 18.1	22.1 ± 12.1*
	F	%	88.3 ± 20.1	45.8 ± 32.4*

Values are mean ± SD of three to six rats. *p < 0.05

*AUC*_*0–12*_ area under the concentration-time curve from time 0 to 12 h, *C*_*max*_ maximum concentration, *k*_*e*_ elimination rate constant, *t*_*1/2*_ half-life, *CL*_*tot*_ total clearance, *V*_*d*_ volume of distribution, *F* bioavailability

### Effects of RFP pretreatment on mRNA expression levels of Mdr1a, Mrp2 and Bcrp in the small intestine of rats

The basal expression of Mdr1a was higher in the middle and lower regions of the intestine than in the upper region, and Mrp2 was more highly expressed in the middle region (Table 3). After four days of pretreatment with RFP, Mdr1a mRNA was significantly increased by 1.5-fold in the middle part of the small intestine, while Mrp2 mRNA was increased by 1.6- and 1.8-fold in the upper and middle parts of the small intestine, respectively. There was no marked change in Bcrp mRNA levels.

**Table 3:** mRNA expression of Mdr1a, Mrp2 and Bcrp in the small intestine with and without RFP

		mRNA expression level (copy/ng RNA)	
		Control	with RFP
Mdr1a	Upper	11,800 ± 6900	14,300 ± 8300
	Middle	22,600 ± 5400	33,600 ± 11700*
	Lower	18,900 ± 7200	27,200 ± 9300
Mrp2	Upper	396 ± 164	633 ± 160*
	Middle	683 ± 231	1250 ± 542*
	Lower	299 ± 90	425 ± 131
Bcrp	Upper	3880 ± 1460	3650 ± 921
	Middle	2620 ± 252	2860 ± 526
	Lower	1250 ± 228	1170 ± 430

Values are mean ± SD of five rats. *p < 0.05

*Mdr1a* multidrug resistance protein 1a, *Mrp2* multidrug resistance-associated protein 2, *Bcrp* breast cancer resistance protein

### Effects of RFP on permeability of LZD in rat small intestine (Ussing chamber technique)

As shown in Table 4, the P_app_ values of inulin, a paracellular marker, in the middle intestine of control rats showed no significant difference between the absorption (mucosal to serosal, m-s; 1.40 ± 1.27 × 10^− 6^ cm/s) and secretory (serosal to mucosal, s-m; 1.08 ± 0.86 × 10^− 6^ cm/s) directions, in accordance with the report by Naruhashi et al. [21]. Table 5 shows the permeability of LY, another paracellular marker, across the upper, middle and lower intestinal tissues. The values of P_app,s-m_, P_app,m-s_ and ER of LY in control rats were in line with those reported by Lin et al. [17]. The value of P_app, s-m_ of Rho123 (4.11 ± 2.85 × 10^− 6^ cm/s) was higher than P_app, m-s_ (2.14 ± 1.21 × 10^− 6^ cm/s). The ER of 1.92 confirmed active efflux transport of Rho123 in intestinal epithelial cells. These data suggest that the Ussing chamber system used here was suitable to evaluate drug permeability, especially focusing on P-gp.

**Table 4:** Apparent permeability coefficient of Rho123 and inulin across the middle intestinal tissues of control rats

	P_app_ (× 10^−6^ cm/s)		
	m-s	s-m	ER
Rho123	2.14 ± 1.21	4.11 ± 2.85	1.92
inulin	1.40 ± 1.27	1.08 ± 0.86	0.771

Values are mean ± SD of three rats

*m-s* mucosal to serosal, *s-m* serosal to mucosal, *ER* efflux ratio, *P*_*app*_ apparent permeability coefficient, *Rho123* rhodamine 123

**Table 5:** Effect of RFP pretreatment on the apparent permeability coefficient of LZD across the intestinal tissues

			P_app_ (× 10^−6^ cm/s)		
			m-s	s-m	ER
Upper	Control	LZD	12.5 ± 5.6	11.6 ± 5.2	0.927
		LY	8.93 ± 4.47	8.41 ± 4.14	0.942
	with RFP	LZD	13.6 ± 5.5	12.1 ± 4.3	0.891
		LY	9.09 ± 4.49	8.59 ± 3.24	0.944
Middle	Control	LZD	9.19 ± 2.27	9.44 ± 3.72	1.03
		LY	6.60 ± 2.90	7.06 ± 3.14	1.07
	with RFP	LZD	11.4 ± 3.4	10.2 ± 2.9	0.898
		LY	7.32 ± 2.84	7.12 ± 2.65	0.973
Lower	Control	LZD	13.2 ± 3.0	10.3 ± 4.5	0.782
		LY	8.84 ± 2.72	6.88 ± 3.77	0.778
	with RFP	LZD	14.3 ± 4.0	12.0 ± 4.2	0.842
		LY	8.85 ± 3.04	8.44 ± 3.89	0.953

Values are mean ± SD of five to six rats

*m-s* mucosal to serosal, *s-m* serosal to mucosal, *ER* efflux ratio, *P*_*app*_ apparent permeability coefficient, *LY* Lucifer yellow

**Fig. 2:**
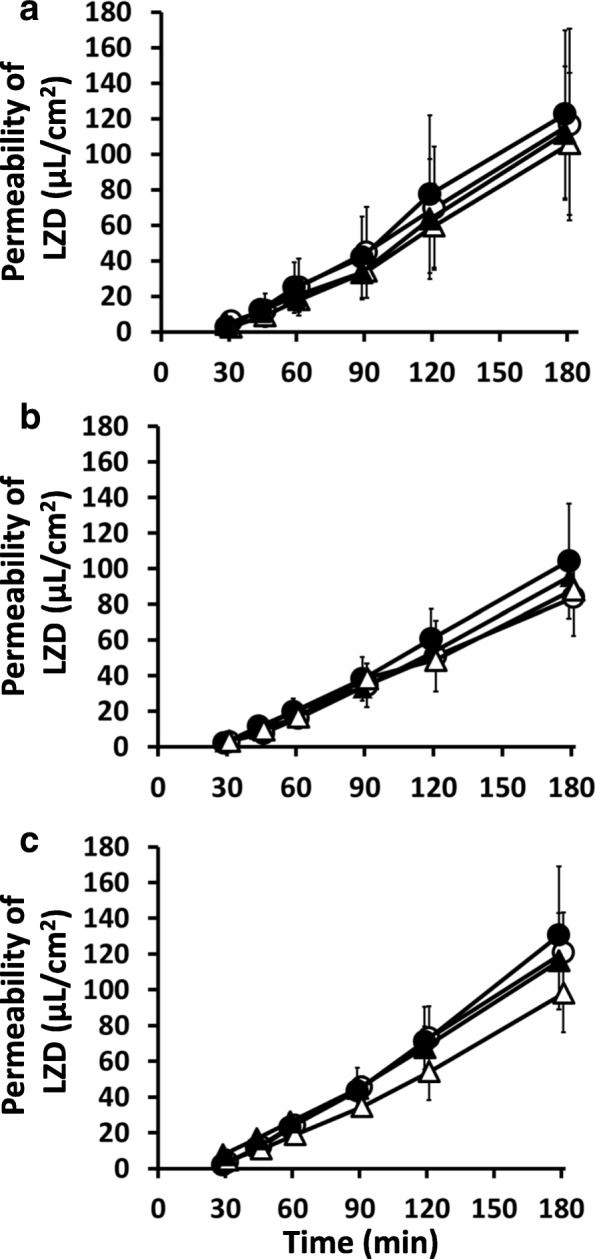
Time course of linezolid permeation with and without RFP in an Ussing chamber experiment. Time course of the mucosal-to-serosal transport (circle) and the serosal-to-mucosal transport (triangle) of linezolid across the rat (**a**) upper, (**b**) middle and (**c**) lower intestinal tissues with (closed) and without (open) RFP pretreatment (10 mg/kg) for four days. Values are expressed as the mean ± SD (*n* = 5–6)

Figure 2 and Table 5 show the time-course of the LZD permeation and the permeability of LZD across the upper, middle and lower intestinal tissues in RFP-pretreated and control rats. In control rats, there was no difference in the P_app_ values of LZD between the m-s and s-m directions in the upper, middle and lower intestine, and the values of ER of LZD showed no difference among all the intestinal regions, regardless of the site-specific expression of Mdr1a and Mrp2 mRNAs. RFP pretreatment did not increase the secretory transport of LZD at any site of the intestine, and also had no effect on the absorptive transport of LZD. The values of ER of LZD at each site of intestine showed no difference from those in control rats. The values of P_app,s-m_, P_app,m-s_ and ER of LY also showed no significant differences between control and RFP-pretreated rats.

## Discussion

LZD plus RFP is a salvage therapy for multidrug-resistant tuberculosis and refractory bone and joint infections due to MRSA, because RFP is effective against bacteria producing a biofilm, while LZD shows good penetration into tissues and no cross-resistance to LZD has been found in strains resistant to other antibiotics [22–24]. Therefore, the use of LZD/RFP therapy has been increasing in recent years. However, there have been a few reports indicating that RFP decreases LZD exposure. This is important, because subtherapeutic levels of LZD present a risk of therapeutic failure and emergence of LZD-resistant strains.

The present clinical study is the first prospective demonstration of DDI between LZD and RFP in Japanese patients. The results of this prospective, open-label, uncontrolled study showed that RFP reduced the dose-normalized trough concentration of orally administered LZD by an average of 65%. This finding is consistent with previous reports. Gandelman et al. found that the C_max_ and AUC values for LZD were reduced by 21% and 32% in healthy subjects when LZD was administered orally after 8 days of pretreatment with RFP [12]. Pea et al. reported that the C_min_ and AUC values for LZD were decreased by 63% and 42% when LZD was administered orally with RFP to patients [13]. We also encountered one patient who was excluded from this clinical study because RFP had been added after the initiation of LZD therapy. His C/D ratio of LZD decreased by about 60% after addition of RFP to LZD therapy, in agreement with a case report by Hoyo et al. [25]. It is important to note that the effect of adding RFP to LZD on the clinical outcome has not been well documented, and trough concentrations of LZD during LZD/RFP therapy have not generally been monitored. However, our results suggest that the decrease of LZD exposure caused by RFP is too large to be disregarded. The DDI between LZD and RFP could contribute to the wide inter-individual variability in LZD exposure. Overall, currently available findings indicate that clinicians should routinely monitor LZD concentrations in patients receiving the combination therapy.

In our animal study, multiple doses of RFP significantly decreased the AUC, C_max_ and F of LZD when LZD was orally administered. In contrast, the pharmacokinetics of intravenously administered LZD showed no change in RFP-pretreated rats. These results of the animal experiments support the idea that LZD concentration is decreased only in the case of oral, but not intravenous, administration of LZD in patients receiving the combination therapy. These results suggested that RFP pretreatment might reduce the permeability of LZD. On the other hand, there are some reports that RFP decreased the trough concentration of LZD in patients even in the case of intravenous LZD administration [26, 27]. The reasons for these apparently conflicting results are unclear, though possible explanations include species differences and differences of clinical conditions, e.g. CRP, because the inflammatory reaction could decrease the expression of some nuclear receptors which in turn control the expression of metabolizing enzymes and transporters [28].

Several mechanisms can be considered to explain the DDI, because RFP induces the expression of CYP3A4 and UDP-glucuronosyl-transferases in liver and intestine, and P-gp and MRP2 in intestine, while it inhibits organic anion transporting polypeptides in liver [29–38]. Multiple doses of RFP decreased exposure to digoxin and nifedipine (substrates of P-gp and CYP3A4/5, respectively) after oral administration of these drugs [36, 38]. On the other hand, the effects were less pronounced after intravenous administration, as was seen in our experiment.

Because preadministration of RFP in rats had no effect on the clearance of LZD administered intravenously, and rat homologues cyp3a1/2 are not induced by RFP [39], we considered that a change of LZD permeability in the intestine could be involved in the reduction of LZD concentration in the case of oral administration. Therefore, we investigated the influence of RFP on mRNA expression of efflux transporters, and we also examined its effect on the intestinal permeability of LZD by means of an Ussing chamber experiment. However, multiple doses of RFP had no effect on the intestinal permeability of LZD, and no site-specificity in the small-intestinal absorption of LZD was found in the Ussing chamber experiment, even though intestinal Mdr1a and Mrp2 are expressed and induced site-specifically. These findings suggest that P-gp and MRP2 in the intestine make little contribution to the pharmacokinetics of LZD. Thus, the decrease of bioavailability of LZD after multiple doses of RFP appears not to be due to a decrease of intestinal permeability. The complicated DDIs with RFP can lead to apparently paradoxical observations in evaluation of the pharmacokinetics of co-administered drugs. Multiple doses of RFP had no effect on the small-intestinal absorption of LZD in the Ussing chamber experiment, even though Mdr1a and Mrp2 are expressed in the intestine. In general, oral bioavailability is calculated as the product of absorption rate, intestinal availability, and liver availability. Our results indicated that RFP had no effect on the absorption rate or intestinal availability of LZD, suggesting that a first-pass effect in the liver may be the major contributor to the DDI between LZD and RFP.

Our study has some limitations. First, although the present study is the first prospective demonstration of DDI between LZD and RFP in Japanese patients, the small sample size in the clinical study limited the power of the statistical analysis. Further study with a larger number of cases will be needed to confirm our findings. Second, our results did not completely exclude the possibility that P-gp might be involved in the intestinal permeability of LZD, because we did not examine the effect of a P-gp inhibitor on the intestinal permeability. Third, it is not known whether coadministered RFP alters the metabolism of LZD, as the urinary excretion of LZD and its metabolites was not evaluated.

## Conclusions

Multiple doses of RFP decreased the AUC, C_max_ and F of orally administered LZD in the case of combined treatment, but had no effect on LZD after intravenous administration in rats. However, RFP did not affect the intestinal absorption of LZD. Further work will be needed to establish the mechanism of the DDI between RFP and LZD.

## Abbreviations

AUC: Area under the concentration-time curve
Bcrp: Breast cancer resistance protein
C/D ratio: Dose-normalized trough concentration
CL_tot_: Total clearance
C_max_: Maximum concentration
C_min_: Trough concentration
CRP: C-reactive protein
CYP: Cytochrome P450
DDI: Drug-drug interaction
eGFR: Estimated glomerular filtration rate
ER: Efflux ratio
F: Bioavailability
Hb: Hemoglobin
HPLC: High performance liquid chromatography
k_e_: Elimination rate constant
LC/MS: Liquid chromatography-mass spectrometry
LY: Lucifer yellow
LZD: Linezolid
Mdr1a: Multidrug resistance protein 1a
Mrp2: Multidrug resistance-associated protein 2
MRSA: Methicillin-resistant *Staphylococcus aureus*
P_app_: Apparent permeability coefficient
P-gp: P-glycoprotein
RFP: Rifampicin
Rho123: Rhodamine 123
t_1/2_: Half-life
TDM: Therapeutic drug monitoring
V_d_: Volume of distribution
